# Development of [^89^Zr]Zr-hCD103.Fab01A and [^68^Ga]Ga-hCD103.Fab01A for PET imaging to noninvasively assess cancer reactive T cell infiltration: Fab-based CD103 immunoPET

**DOI:** 10.1186/s13550-023-01043-9

**Published:** 2023-11-20

**Authors:** Xiaoyu Fan, Marta A. Ważyńska, Arjan Kol, Noemi Perujo Holland, Bruna Fernandes, Sander M. J. van Duijnhoven, Annechien Plat, Hans van Eenennaam, Philip H. Elsinga, Hans W. Nijman, Marco de Bruyn

**Affiliations:** 1 Department of Nuclear Medicine and Molecular Imaging, University of Groningen, University Medical Center Groningen, Groningen, The Netherlands; 2 Department of Obstetrics and Gynecology, University of Groningen, University Medical Center Groningen, Groningen, The Netherlands; 3 Aduro Biotech Europe, Oss, The Netherlands

**Keywords:** CD103, Biomarker, ImmunoPET, ^89^Zr, ^68^Ga

## Abstract

**Background:**

CD103 is an integrin specifically expressed on the surface of cancer-reactive T cells. The number of CD103+ T cells significantly increases during successful immunotherapy and might therefore be an attractive biomarker for noninvasive PET imaging of immunotherapy response. Since the long half-life of antibodies preclude repeat imaging of CD103+ T cell dynamics early in therapy, we therefore here explored PET imaging with CD103 Fab fragments radiolabeled with a longer (^89^Zr) and shorter-lived radionuclide (^68^Ga).

**Methods:**

Antihuman CD103 Fab fragment Fab01A was radiolabeled with ^89^Zr or ^68^Ga, generating [^89^Zr]Zr-hCD103.Fab01A and [^68^Ga]Ga-hCD103.Fab01A, respectively. In vivo evaluation of these tracers was performed in male nude mice (BALB/cOlaHsd-Foxn1nu) with established CD103-expressing CHO (CHO.CD103) or CHO-wildtype (CHO.K1) xenografts, followed by serial PET imaging and ex vivo bio-distribution.

**Results:**

[^89^Zr]Zr-hCD103.Fab01A showed high tracer uptake in CD103+ xenografts as early as 3 h post-injection. However, the background signal remained high in the 3- and 6-h scans. The background was relatively low at 24 h after injection with sufficient tumor uptake. [^68^Ga]Ga-hCD103.Fab01Ashowed acceptable uptake and signal-to-noise ratio in CD103+ xenografts after 3 h, which decreased at subsequent time points.

**Conclusion:**

[^89^Zr]Zr-hCD103.Fab01A demonstrated a relatively low background and high xenograft uptake in scans as early as 6 h post-injection and could be explored for repeat imaging during immunotherapy in clinical trials. ^18^F or ^64^Cu could be explored as alternative to ^68^Ga in optimizing half-life and radiation burden of the tracer.

**Supplementary Information:**

The online version contains supplementary material available at 10.1186/s13550-023-01043-9.

## Introduction

Cancer immunotherapy has revolutionized the field of immunology and oncology. At its core, immunotherapy relies on generating or augmenting adaptive immune responses against antigens preferentially or selectively expressed in cancer cells [1]. This is best exemplified by monoclonal antibody therapies that block the immune checkpoint programmed death-1 (PD-1) or its ligand (PD-L1), and their exquisite activity in patients with a high mutation burden [2]. Nevertheless, the majority of patients do not respond to immune checkpoint therapy and early response biomarkers that can guide treatment decision making are urgently needed.

One of the indicators of successful immunotherapy across tumor types is an increase in the number of tumor-infiltrating lymphocytes (TILs) [3]. TILs in tumor lesions are correlated with greater immunological responses; therefore, they represent attractive biomarkers for monitoring immunotherapy. However, not every T cell within a tumor is involved in the anti-cancer immune response [4]. In recent years, we and others have refined the definition of cancer-reactive TILs [5]. Across different phenotypes of T-lymphocytes, tissue-resident memory-like cells (T_RM_-like cell) have emerged as a subset with a prognostic value across different malignancies. These noncirculating memory T cells (T_RM_-like cells) are linked to an integrated immune response and a favorable outcome of immune checkpoint blockade therapy in patients [6–8]. A primary cell surface marker of CD8+ T_RM_-like cells is the αE subunit of the αEβ7 integrin complex, commonly known as CD103. This integrin mediates retention of lymphocytes in peripheral tissues by binding to E-cadherin expressed on epithelial cells [8]. Recent work has shown that in melanoma, lung and esophageal cancer patients, the number of CD103+ TILs was significantly increased during immunotherapy in responding lesions in comparison with lesions of treatment-naive patients and nonresponders [9, 10]. Thus, CD103 is an interesting biomarker for the assessment of cancer reactive T cell infiltration.

The current standard for determination of CD103+ cell infiltration is immunohistochemistry staining (IHC) on tissue biopsies. However, biopsies have several disadvantages. Among those shortcomings are tumor heterogeneity within and between lesions, poor accessibility of lesions, and sampling errors. In order to obtain accurate information about CD103+ T_RM_ load in all tumor lesions, noninvasive whole-body imaging techniques can be applied. The most suitable technique in the clinical setting is positron emission tomography (PET), a diagnostic tool for functional/molecular imaging. PET offers high sensitivity and fast imaging time and requires small amounts of molecular probe to be used in comparison with other imaging techniques [11]. Moreover, PET allows for repetitive and noninvasive clinical assessment. The possibility of imaging signal quantification is ideally suited for determining whole-body T_RM_ load using radiolabeled antibody fragments (Fabs). For monoclonal antibodies, zirconium-89 (^89^Zr; *t*_*1/2*_ = 78.4 h) is a suitable isotope for radiolabeling, as its physical half-life matches the time mAbs which require for achieving optimal target-to-background signals. In the case of antibody fragments, radioisotopes with a shorter half-life could also be applicable, given that Fabs are smaller in size and thus have a faster biodistribution [12]. An alternative radioisotope that was successfully used in the past for antibody-based tracer labeling is gallium-68 (^68^Ga; *t*_*1/2*_ = 67.7 min) [13]. Recently, a first-in-human PET imaging study with ^89^Zr-labeled atezolizumab (anti-PD-L1) showed dependency of tracer uptake in response to atezolizumab treatment [14]. Noninvasive PET imaging of T cells has also been described in mouse models and clinical trials using markers such as CD3 and CD8 [15, 16]. Here, we generated [^89^Zr]Zr-hCD103.Fab01A and [^68^Ga]Ga-hCD103. Fab01A tracers that specifically recognize human CD103 for noninvasive immuno-PET imaging of T cell infiltration in human cancers as a potential biomarker for effective anti-cancer immune responses.

## Results

### Tracer development and quality control of [^89^Zr]Zr-hCD103.Fab01A and [^68^Ga]Ga-hCD103.Fab01A

**Fig. 1:**
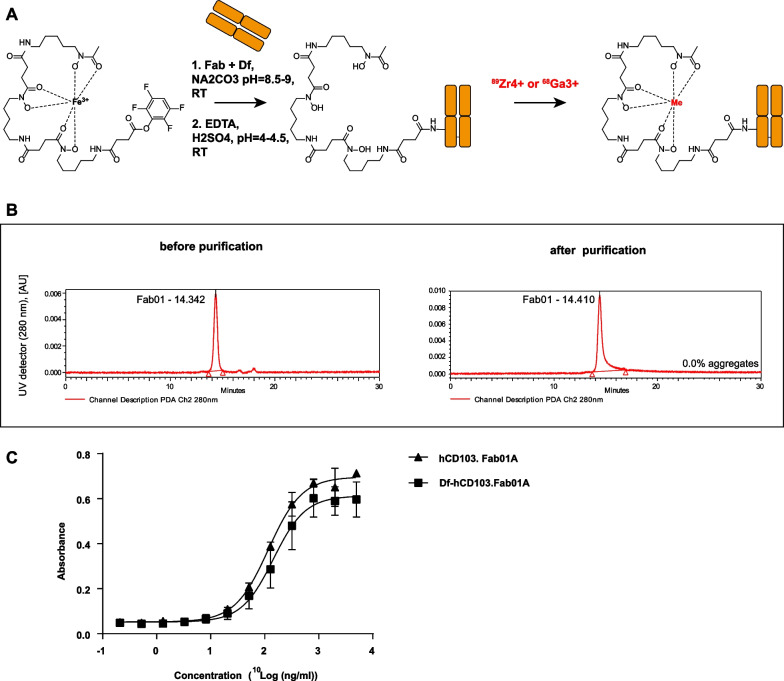
Tracer development **A** TFP-N-Suc-desferal-Fe chelator conjugation and ^89^Zr/^68^Ga radiolabeling reaction scheme. **B** SE-UPLC chromatograms of hCD103.Fab01A conjugated with the chelator TFP-N-Suc-desferal-Fe using UV detection at 280 nm (protein). **C** Binding ability of Df-hCD103.Fab01A and hCD103.Fab01A to CHO.CD103 was assessed by cell-based ELISA

A Fab fragment was recombinantly produced from the heavy and light chain sequences of a previously developed antihuman CD103 antibody clone 01A (hCD103.Fab01A). The conjugation with TFP-N-Suc-Desferal-Fe (Df, ABX GmbH, Hamburg, Germany) and radiolabeling procedure of hCD103.Fab01A are summarized in Fig. 1A. Conjugation was performed as previously described using 4 molar excess of TFP-N-Suc-Df-Fe, followed by Fe^3+^ transchelation using EDTA and purification using Vivaspin ultrafiltration spin columns. The conjugation efficiency was 64.7% and was calculated based on the percentage of area under the curve (AUC) at 430 nm wavelength corresponding to retention time of hCD103.Fab01A on size exclusion ultra-performance liquid chromatography (SE-UPLC). Chelator to Fab fragment ratio was determined to be 2.6:1 (Fig. 1B, Additional file 1: Fig. S1E). After the purification, conjugates showed a mono-peak at 280 nm wavelength on SE-UPLC (Fig. 1B).The binding ability of the Df-conjugated-hCD103 compared to the unmodified antibody was determined by cell-based ELISA using CD103/β7 transfected CHO cells, which showed a slight decrease after the conjugation (log EC50: 2.07 ng/mL for hCD103.Fab01A, 2.16 ng/mL for Df- hCD103.Fab01A) **(**Fig. 1C).

Gallium-68 labeling of hCD103.Fab01A -N-suc-Df was achieved by following adapted literature procedures described by Oroujeni [13]. Due to the fragile nature of the Fab fragment, the labeling temperature was kept at 25 °C. HEPES buffer was used as in zirconium-89 labeling, which yielded in a conversion of over 90%. Conversion was measured by iTLC, where the radiolabeled Fab fragment was retained on the baseline, and unlabeled ^68^Ga was complexed by citric acid and had no retention on the iTLC (Additional file 1: Fig. S1B). Ratio between activity and amount of hCD103.Fab01A-N-suc-Df was also investigated and determined to be the best at 1300 MBq ^68^Ga per 1 mg of hCD103-N-suc-Df (Additional file 1: Fig. S1C). Repeated labeling gave an optimal time point of 15 min reaction time (Additional file 1: Fig. S1A). If purification was needed (RCP < 90%), a miniPD-10 desalting column was used (Additional file 4: Table S1). Due to protein retention on the column, a new concentration was determined by UPLC (Additional file 1: Fig. S1D).

Based on the CD103 human mAb tracer development, 300 MBq/mg was selected for in vivo studies for zirconium-labeled Fab tracer [17]. Generally, 1 h of labeling was sufficient to obtain RCP > 95%; however, if the desired purity was not reached, the reaction was left to proceed longer. After labeling, as determined by SE-UPLC, [^89^Zr]Zr hCD103.Fab01A showed a single Fab fragment peak with a HEPES buffer peak at 280 nm by UV detector and a single peak with a small peak of free ^89^Zr (AUC < 5%) by radioactivity detector. Radiochemical purity (RCP) after labeling was determined by the TCA test, and the average RCP is above 95% without further purification (Additional file 1: Fig. S1F, Additional file 4: Table S1).

### In vitro and ex vivo stability of [^89^Zr]Zr-hCD103.Fab01A and [^68^Ga]Ga-hCD103.Fab01A

**Fig. 2:**
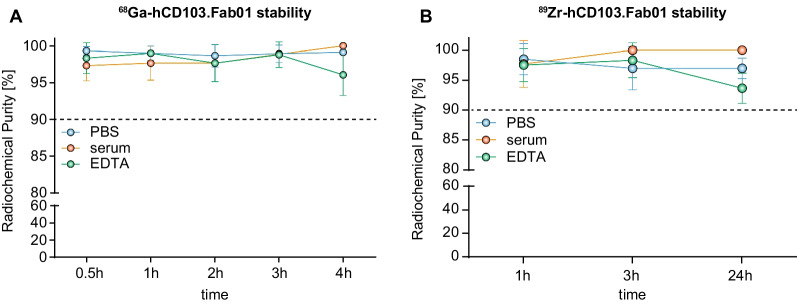
Tracer stability in PBS, serum and EDTA challenge of **A** [^68^Ga]Ga-hCD103.Fab01A and **B** [^89^Zr]Zr-hCD103.Fab01A

In vitro stability tests of the tracers at room temperature were performed in PBS and in the presence of 1000-fold molar excess of EDTA, whereas ex vivo stability was conducted in serum. As determined by iTLC, the results of the in vitro and ex vivo stability test demonstrated that ^89^Zr- and ^68^Ga-labeled hCD103.Fab01A are stable in PBS, serum and under EDTA challenge for 4 h in the case of the ^68^Ga-labeled product (mean = 99%, 100%, 96%, respectively) (Fig. 2A) and up to 24 h for ^89^Zr-labeled product (mean = 97%, 100%, 94%, respectively) (Fig. 2B). Different time points were chosen for the tests according to the different half-lives of the radionuclides.

### In vitro binding ability of [^89^Zr]Zr-hCD103.Fab01A and [^68^Ga]Ga-hCD103.Fab01A

**Fig. 3:**
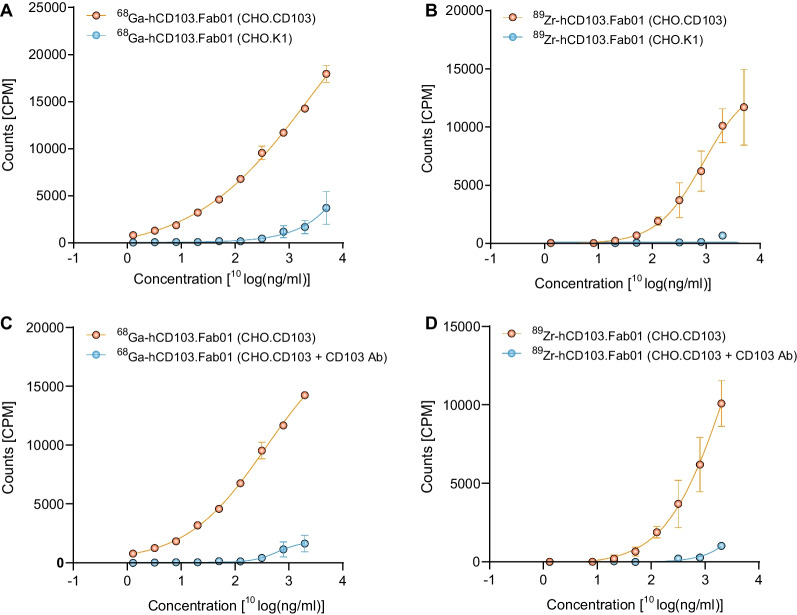
In vitro specificity of [^68^Ga]Ga-hCD103.Fab01A and [^89^Zr]Zr-hCD103.Fab01A **A**, **B** Binding of [^68^Ga]Ga-hCD103.Fab01A and [^89^Zr]Zr-hCD103.Fab01A to CHO.CD103 cells and CHO-K1 wild-type cells. Radioactivity was measured using a gamma counter. **C**, **D** Blocking assay with [^68^Ga]Ga-hCD103.Fab01A and [^89^Zr]Zr-hCD103.Fab01A to CHO.CD103 cells. Radioactivity was measured using a gamma counter

The specificity of [^68^]Ga-hCD103.Fab01A and [^89^Zr]Zr-hCD103.Fab01A was tested using CHO.K1 cells transfected with alphaE/beta7 integrin (CD103/β7). Both tracers, ^68^Ga- and ^89^Zr-labeled hCD103.Fab01A, exhibited specific binding to CHO.CD103 cells (log EC50: 2.94 ng/mL for [89Zr]Zr-hCD103.Fab01A, 3.41 ng/mL for [68Ga]Ga-hCD103.Fab01A) (Fig. 3A, B). Some unspecific binding to parental CHO.K1 cells was observed at higher concentrations of the tested tracers. Blocking studies by preincubation of CHO.CD103 and CHO.K1 with commercially available competing hCD103 mAb demonstrated that both tracers bind specifically to CD103 (Fig. 3C, D).

### PET imaging of [^89^Zr]Zr-hCD103.Fab01A and [^68^Ga]Ga-hCD103.Fab01A

To study whether [^89^Zr]Zr-hCD103.Fab01A and [^68^Ga]Ga-hCD103.Fab01A PET imaging can visualize human CD103 in vivo*,* male nude mice (BALB/cOlaHsd-Foxn1nu) were subcutaneously inoculated with CHO.CD103 or CHO.K1 in the shoulder/neck area. Xenografts were allowed to grow to at least 200 mm^3^. Both CHO.K1 and CHO.CD103 cell lines grew reproducibly in vivo (Additional file 2: Fig. S2A) and maintained CD103 expression ex vivo, as shown by immunohistochemical staining (Additional file 2: Fig. S2B).

For microPET imaging, xenograft-bearing mice (*n* = 4 per group) were injected intravenously via the penile vein with [^89^Zr]Zr-hCD103.Fab01A (8.6 ± 0.4 μg, 2.8 ± 0.2 MBq) or [^68^Ga]Ga-hCD103.Fab01A (7.1 ± 0.6 μg, 9.9 ± 1.0 MBq). For [^89^Zr]Zr-hCD103.Fab01A, microPET scans were made 3, 6 and 24 h post-injection based on previous studies with anti-CD103 mAb tracers [17]. For [^68^Ga]Ga-hCD103.Fab01A, microPET scans were performed at 3 h post-injection due to the short half-life of ^68^Ga.

**Fig. 4:**
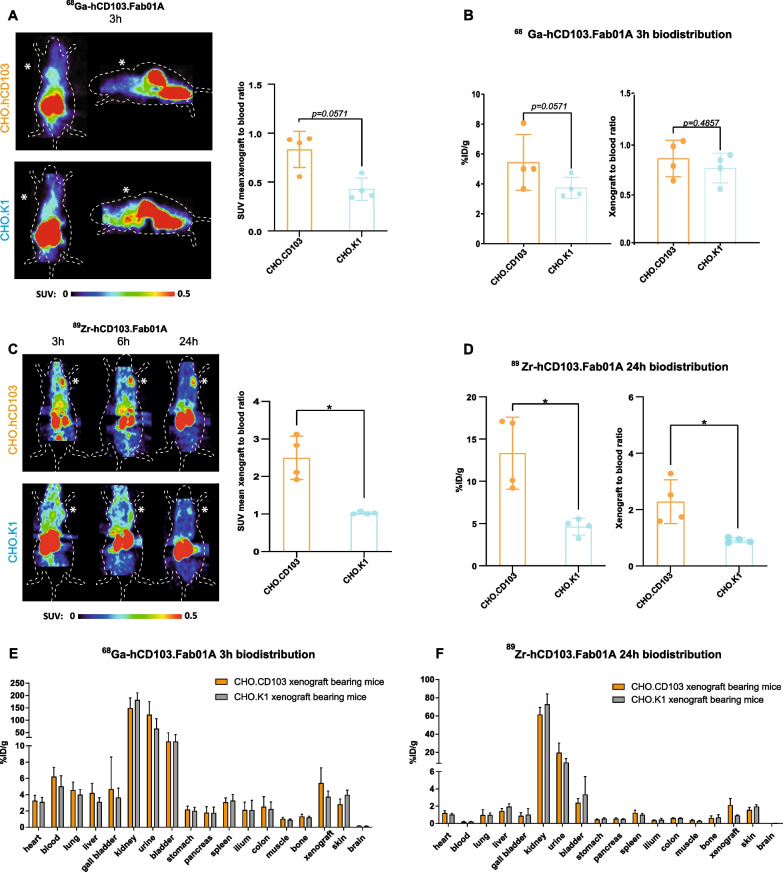
In vivo [^89^Zr]Zr-hCD103.Fab01A and [^68^Ga]Ga-hCD103.Fab01A microPET imaging **A** Representative coronal and sagittal [^68^Ga]Ga-hCD103.Fab01A PET scans, 3 h post-tracer injection in CHO.CD103 (top panel) and CHO.K1 xenograft mice (bottom panel). Xenograft area marked with asterix. In vivo [^68^Ga]Ga-hCD103.Fab01A xenograft-to-blood ratio, 3 h post-tracer injection, are expressed as SUV_mean_. **B** Ex vivo xenograft uptake (%ID/g) and xenograft-to-blood ratio of ^6^[^68^Ga]Ga-hCD103.Fab01A, 3 h post-tracer injection. **C** Representative coronal [^89^Zr]Zr-hCD103.Fab01A PET scans, 3, 6 and 24 h post-tracer injection in CHO.CD103 (top panel) and CHO.K1 xenograft mice (bottom panel). Xenograft area marked with asterix. In vivo [^89^Zr]Zr-hCD103.Fab01A xenograft-to-blood ratio, 24 h post-tracer injection, are expressed as SUV_mean_. **D** Ex vivo xenograft uptake (%ID/g) and xenograft-to-blood ratio of [^89^Zr]Zr-hCD103.Fab01A, 24 h post-tracer injection. *, *p* < 0.05. **E**–**F** Ex vivo tissue uptake (%ID/g) of [^68^Ga]Ga-hCD103.Fab01A (**E**) and [^89^Zr]Zr-hCD103.Fab01A (**F**) in CHO.CD103 and CHO.K1 xenograft bearing mice

PET scans of CHO.CD103 xenograft bearing mice with [^68^Ga]Ga-hCD103.Fab01A showed an increased tumor uptake compared with the control group (CHO.K1 tumor bearing mice), which was further confirmed by SUV_mean_ quantification (mean xenograft-to-blood ratio: 0.83 vs 0.43, p = 0.057, Fig. 4A, Additional file 3: Fig. S3). Ex vivo biodistribution analysis after 3 h showed a trend toward higher [^68^Ga]Ga-hCD103.Fab01A uptake in CHO.CD103 xenografts in comparison with CHO.K1 xenograft (mean %ID/g: 5.4 vs 3.7, p = 0.057, Fig. 4B, left panel). The tumor-to-blood ratio of [^68^Ga]Ga-hCD103.Fab01A in CHO.CD103 was only slightly higher than the control group and not significant (mean 0.9 vs 0.8, p = 0.4857, Fig. 4B, right panel).

As shown in the scans (Fig. 4C, Additional file 3: Fig. S3), CHO.CD103.

xenograft mice injected with [^89^Zr]Zr-hCD103.Fab01A showed high tracer uptake within the xenograft as early as 3 h after injection. However, the background signal remained high in the 3 h and 6 h scans, so the background-to-signal ratio was not optimal. At 24 h, the background was relatively low, while xenograft uptake remained very pronounced. The SUVmean of 24 h post-injection compared CHO.CD103 to CHO.K1 showed a significant higher uptake in CHO.CD103 xenograft (mean xenograft-to-blood ratio 2.50 vs 1.03, *p* = 0.0286) (Fig. 4C). Ex vivo biodistribution analysis after 24 h showed significantly higher [^89^Zr]Zr-hCD103.Fab01A specific xenograft uptake of CHO.CD103 than CHO.K1 (mean %ID/g: 2.28 vs 0.91, *p* = 0.0286; Fig. 4D, right panel). Ex vivo xenograft-to-blood ratio of [^89^Zr]Zr-hCD103.Fab01A was significantly higher in CHO.CD103 than in CHO.K1 (mean 13.33 vs 4.63, *p* = 0.0286; Fig. 4D, left panel).

Higher tracer uptake in the kidney, urine, and bladder was observed in both tracers (Fig. 4A, C, E, F). Immunohistochemical staining of the kidney for CD103 showed no CD103 expression in the kidney suggesting that the high uptake in the kidney was not related to specific tracer uptake but rather due to renal clearance (Additional file 2: Fig. S2B). In addition, we observed higher background signals in the 3 h [^68^Ga]Ga-hCD103.Fab01A scan and ex vivo analysis results (Fig. 4A, C, E, F) compared to the 24 h [^89^Zr]Zr-hCD103.Fab01A analysis.

## Discussion

To date, PET imaging of T cells has been described in preclinical models targeting T cell surface markers CD3, CD4 and CD8, and uptake was correlated with response to immunotherapy [15, 16, 18]. The most potent effectors of the antitumor immune response are CD8+ cytotoxic T cells. Therefore, CD8+ T cell imaging is currently considered to be the most promising tool for the early identification of immune surveillance function [18]. In clinical trials, anti-CD8 imaging agents are under investigation examining patients before, during and after treatment with checkpoint inhibitors (NCT04029181, NCT03802123). However, the presence of CD8+ TILs in tumor tissue does not mean that these TILs are functional. A prominent feature of immune escape is T cell depletion. Instead of CD8, CD103^+^ resident-like tumor-infiltrating T cells have been reported as a subtype of tissue-resident memory T cells (T_RM_) that have been found in the tumor microenvironment [19, 20] and have been shown to have a prognostic effect across multiple types of solid cancers, including cervical cancer [21], head & neck squamous cell carcinoma [22], lung and bladder cancer [23], cholangiocarcinoma [24], gastric cancer [25], ovarian cancer [26], esophageal squamous cell carcinoma [27], colorectal cancer [28], and melanoma [20]. Furthermore, when we compared the preclinical performance of the [^89^Zr]Zr-hCD103.Fab01A tracer with that of the [^89^Zr]Zr-DFO-CD8a F(ab)'2 tracer[29] in syngeneic mice, both tracers showed a comparable uptake in the xenografts at the selected scan time point ([^89^Zr]Zr-hCD103.Fab01A vs [^89^Zr]Zr-DFO-CD8a F(ab)’2, mean %ID/g: 2.28 vs 2.44, mean xenograft/tumor-to-blood ratio 13.33 vs 14.69). As such, CD103+ TRM cells represent an interesting biomarker for evaluating responses to immune checkpoint inhibitors and potentially discriminate responding vs. nonresponding patients, by comparing pre- and on-treatment densities of CD103+ TRM. Herein, the use of a noninvasive imaging modality such as PET offers significant advantages over classical biopsy-based approaches that are hampered by the requirement for accessible lesions and tumor heterogeneity.

The CD103+ TRM imaging strategy proposed here will have applications beyond those described for cancer. CD103+ TRM cells are implicated in a number of auto-immune diseases such as inflammatory bowel disease and multiple sclerosis. CD103+ TRM cells have also been shown to play a significant role in the rejection of transplanted organs, and strategies that deplete CD103+ cells have shown therapeutic success in preclinical models of these disease indications. Like proposed for cancer, CD103 imaging may help diagnose patients with diseases mediated by CD103+ TRM, which are frequently difficult to assess through biopsy-based methods. CD103 imaging may also help monitor therapy responses, discriminate responders from nonresponders and help guide treatment decision making in these indications. Finally, CD103 is expressed on a series of leukemic cancers, such as hairy cell leukemia [30] and may be used for diagnosis and monitoring of disease progression in response to therapy.

Recent preclinical studies have demonstrated that the use of mAbs for T cell imaging can impair T cell function, despite being administered at low doses [14, 31]. These functional effects of mAbs are likely due to their bivalent nature and interaction with specific Fc receptors. As a result of these interactions, full-length mAbs can trigger antigen crosslinking and cell- or complement-mediated effector functions. For mAbs used therapeutically in parallel to imaging, these considerations are mute. However, the requirement for parallel use of antibodies as therapeutic and imaging agents limits the potential for noninvasive imaging of clinically relevant immune subsets that do not directly represent a therapeutic target, such as CD103+ TRM cells. Here, we demonstrate that Fab derivation of a previously developed anti-CD103 imaging antibody could be used to side-step these issues while maintaining the previously described high target-to-background ratios, high target site selectivity and a high sensitivity of this mAb. While highly promising, care should be taken with regards to the generalization of our observations as the mAbs should possess sufficient single arm binding affinity to allow derivation as a Fab without compromising binding activity. Although not discussed extensively here, several of the previously developed anti-CD103 mAbs failed to exert sufficient binding affinity to allow use as Fabs as imaging agents [17].

Due to the relatively large molecular size of the full-length mAbs, their serum half-life is often more than ten days. Therefore mAbs need to be labeled by radionuclides with a longer half-life, such as ^89^Zr (*t*_1/2_ = 78.4 h) [32]. Fabs, as a fragment of the mAb, have a relatively short serum half-life of 12–20 h compared to full-length mAbs [33], which implies rapid clearance from the blood and nonspecific compartments. With its shorter serum half-life, Fabs could potentially be labeled with the shorter-lived radionuclides. However, uptake of the ^68^Ga-labeled CD103 Fab tracer, was not statistically significant and a considerable amount of background was present. In further studies, other isotopes such as ^64^Cu (*t*_1/2_ = 12.7 h) or ^18^F (*t*_1/2_ = 110 min) may be more suitable for Fab imaging. Labeling with ^18^F could be highly interesting due to usage of aluminium-[^18^F] fluoride with the RESCA chelator. As previously demonstrated [34], RESCA allows for the straightforward radiolabeling of proteins in mild conditions, crucial for Fabs.

In conclusion, we developed a potent high-affinity Fab tracer to image CD103+ TRM cells as a novel tool in monitoring of immunotherapy. However, optimization of the half-life of the applied radionuclide is required.

## Conclusions

This study establishes proof-of-concept for the noninvasive assessment of tumor-resident memory T cells (TRM) through Fab-based CD103 PET imaging. In vitro, we leveraged a previously developed high-affinity anti-CD103 mAb clone to generate a Fab with high single-arm binding affinity. Next, we compared [^68^Ga]Ga- and [^89^Zr]Zr-labeled Fabs for anti-CD103 immuno-PET and demonstrated that this approach can be used to visualize CD103-positive cells with high target-to-background ratios, high target site selectivity and a high sensitivity in hCD103-positive xenografts.

## Materials and methods

### Recombinant molecules and antibodies

All antibodies used in this study are listed in Additional file 4: Table S2.

### Cell lines culturing

Chinese hamster ovary (CHO)-K1 cells were obtained from the American Type Culture Collection (ATCC). Cells were quarantined until screening for microbial contamination, and mycoplasma was performed and proved to be negative. Cells were grown in DMEM/F-12, GlutaMAX™ Supplement + 5% FCS + 25 mM HEPES and incubated in a humidified atmosphere with 5% CO_2_ at 37 °C.

CD103/β7 expressing CHO clones were previously reported [17] and generated by nonliposomal transfection (FuGENE) of pcDNA3.1+_Hygro encoding ITGB7_HUMAN (uniProtKB #P26010) and pCI-neo encoding ITGAE_HUMAN (uniProtKB #P38570) plasmids (GeneArt/ThermoFisher Regensburg, Germany).

### Generation of hCD103 Fab fragment

Anti-hCD103 Fab candidates were produced by ImmunoPrecise. Synthetic vectors encoding for the DNA sequences of the VH and VL domains of previously described antibody hCD103.01A was synthesized and subsequently cloned into ImmunoPrecise’s human IgG1-Fab-K vector and human kappa light chain vector, respectively, followed by transfection of HEK293 cells. Fab fragments from harvested supernatants were purified by endotoxin-free purification using CaptureSelect IgG-CH1 affinity matrix. The molecular weight (MW) of the hCD103.01A is 50 kDa. Fab concentrations were quantified using spectrophotometry and Fab purity was assessed by SDS-PAGE and HP-SEC. Endotoxin levels were determined by LAL assay. Previous work on anti-CD103 mAb tracer development [17] showed that among 6 mAb candidates, clone 01A have the strong binding to the CD103+ CD8+ T cell subpopulation in tumor digests, nonoverlapping binding epitopes and differential CD103 blocking properties. Therefore, for further Fab tracer development we selected Fab 01A.

### [^89^Zr]Zr-hCD103.Fab01A and [^68^Ga]Ga-hCD103.Fab01A tracer development and quality control

TFP-N-Suc-desferal-Fe (Df, ABX GmbH, Hamburg, Germany) was conjugated with a fourfold molar excess to lysine residues of hCD103.Fab01A based on the previous study [17]. The identity and purity of Df-Fab conjugate was determined by size exclusion ultra-performance liquid chromatography (SE-UPLC). The Waters ACQUITY SE-UPLC system was equipped with a dual wavelength absorbance detector, in-line radioactivity detector and UPLC column: TSK-GEL G3000SWXL column (JSB, Eindhoven, Netherlands) or BioSep SEC-s3000, (LC column 300 × 7.8 mm, Phenomenex, Netherlands). The conjugate was aliquoted and frozen.

^89^Zr-labeling was performed as described earlier [31] using clinical-grade [^89^Zr]Zr- oxalate (PerkinElmer, Amsterdam, The Netherlands). Radiochemical purity (RCP) was checked by trichloroacetic acid (TCA) precipitation test. The quality control concerned with aggregation and fragmentation of both conjugates and tracer was checked by size exclusion ultra-performance liquid chromatography (SE-UPLC). Radioactivity during the labeling was measured by the dose calibrator (VDC-505, Comecer, Netherlands).

For TCA test, one microliter (1 μL) of the final tracer products was added to 1 ml of 0.5% human serum albumin (HSA). Additionally, 1 mL of TCA was added and mixed, centrifuged the mixture at 3000 rpm for 10 min. After centrifugation, 1 mL of the supernatant was carefully transferred to another tube, leaving 1 ml of the supernatant and the pellet in the original tube. The radioactivity in both tubes was then measured using a 2480 Wizard Detector Gamma Counter (PerkinElmer, Netherlands).

^68^Ga was obtained by fractionated elution of the < 9 month old GMP ^68^Ge/^68^Ga generator (Eckert and Ziegler, Berlin, Germany) with 0.1 M HCl. The eluate with the highest radioactivity concentration was used for labeling. Briefly, the [^68^Ga]GaCl_3_ (5–10 mL, 600–1000 MBq) was concentrated on a PS-H^+^ cartridge and eluted with a 200 μL 5 M NaCl solution. 80 MBq of [^68^Ga]GaCl_3_ (100–200 μL) was added to into a reaction vial containing N-Suc-Df‐hCD103.Fab01 solution (30–60 μg, 5 mg/mL) mixed with 100 μL of HEPES buffer (1 M, pH 8). The reaction mixture was allowed to react for 15 min at room temperature while being thoroughly vortexed. If RCP did not reach 90%, purification was performed with pre-conditioned (8 ml of PBS) PD10 miniTrap G-25 Sephadex resin size exclusion column (Cytiva, MA, USA) by PBS fractioned elution (100 μL). The labeling yield and radiochemical purity of labeled conjugates were measured using the instant thin-layer chromatography (ITLC) (ITLC-SG, Agilent Technologies, Santa Clara, CA, USA). The strips were developed with 0.05 M citric acid. Distribution of the radioactivity among the strips was measured on GE Amersham Typhoon Scanner using phosphorus plates and ImageQuant TL 1D software for data processing (both GE Healthcare Life Science, USA).

### Cell based ELISA

To compare the binding ability of conjugate and the antibody, the following experiment was conducted. One day before the experiment, CD103/β7 transfected CHO cells (30 000 cells/well in 50 μL) were seeded in 96-well plates. Subsequently, serial dilutions of CD103 Fabs, CD103 Fabs-N-Suc-Df conjugate and isotype controls were added to each well of a 96-well plate and incubated for 1 h at 37 °C. The final total volume in each well was 100 μL. Wells were washed with PBS and incubated with rabbit anti-mouse/IgG-HRP (1:4000, Dako) for 1 h at 37 °C. Next wells were washed with 200 μL PBS and 100 μL of TMB substrate (KPL) was added. The color reaction was stopped by adding 1 M HCl solution and the absorbance was measured by a microplate reader (Thermo Scientific).

### In vitro and ex vivo stability of the tracers

In vitro stability of [^89^Zr]Zr-hCD103.Fab01A and [^68^Ga]Ga-hCD103.Fab01A was tested in PBS and in the presence of 1000-fold molar excess of EDTA (ethylenediaminetetraacetic acid), whereas ex vivo stability in human serum. After purification, samples of freshly labeled conjugate (5.9 µg, 50 µL) were mixed with EDTA (64 µg, 2.5 mg/mL in 50 µLPBS). For PBS and serum stability, samples were mixed with equal volumes (100 µL). For [^89^Zr]Zr-hCD103.Fab01A samples were measured after 1, 3, and 24 h. For [^68^Ga]Ga-hCD103.Fab01A samples were measured after 0.5, 1, 2, 3, and 4 h. RCP of samples submitted for stability was quantified using iTLC strips. The strips were developed and measured as described previously. The experiment was performed in triplicate.

### In vitro binding ability of the tracers

To test the in vitro binding ability of both tracers to CHO.CD103 cell and CHO.K1 cell, the following experiment was conducted. CHO.CD103 and CHO.K1 cells were transferred on the day of experiment to 4 ml plastic tubes (250 000 cells/tube in 100 µL of 2% FCS in PBS). A serial dilution of tracer was added to tubes to achieve 10 different concentrations (0–5000 ng/mL). After 1-h incubation at 37 °C, cells were washed thrice with PBS + 2% FBS (2 ml) and bound [^68^Ga]Ga- or [^89^Zr]Zr-hCD103.Fab01A was measured in Wizard Detector Gamma Counter. For blocking studies, CHO.CD103 cells were transferred on the day of experiment to 4-ml plastic tubes (250 000 cells/tube in 100 µL of 2% FCS in PBS) and set of samples were blocked using 1µL CD103 antibody (10 µg/mL, mouse antihuman CD103, clone Ber-ACT8, BD Bioscience) for 1 h before experiment. Afterward, tracer, in 10 different concentrations (0–2500 ng/mL), was added to the samples, incubated for 1 h at 37 °C, and cells were washed thrice with PBS + 2% FBS. Bound [^68^Ga]Ga/ [^89^Zr]Zr-hCD103.Fab01A was measured in Wizard Detector Gamma Counter.

### Animal study

Animal experiments were planned and performed under approval of the Institutional Animal Care and Use Committee of the University of Groningen in agreement with EU Directive 2010/63/EU (IvD: 16395–01-019).

In vivo studies were performed with male nude mice (BALB/cOlaHsd-Foxn1nu, Envigo, The Netherlands, 6–8 weeks) inoculated subcutaneously with CHO.K1 or CHO.CD103 xenografts (5*10^6^ in 300 µL 1:1 PBS and high growth factor Matrigel (BD Biosciences, Breda, The Netherlands)). In order to be visible under PET scans, tracer injection should be performed at least when the xenograft is at least 200 mm^3^.

For microPET imaging, mice (*n* = 4) were injected intravenously (iv) with [^89^Zr]Zr-hCD103.Fab01A and [^68^Ga]Ga-hCD103.Fab01A, respectively. For mice injected with [^89^Zr]Zr-hCD103.Fab01A, scans were made 3, 6 and 24 h post-injection using a Focus 220 PET scanner (CTI Siemens), followed by ex vivo biodistribution analysis after the final scan. For mice injected with [^68^Ga]Ga-hCD103.Fab01A, scan was only made at 3 h post-injection (pi) due to the short half-life of the ^68^Ga (*t*_1/2_ = 68 min). During the scan, anesthesia was induced and maintained by the administration of a mixture of isoflurane (2.5–3.5%), oxygen, and medical air.

PET data were reconstructed into a static image, and in vivo quantification was performed using AMIDE (v1.0.4, Stanford University, Stanford, CA, USA). MicroPET data are presented as mean standardized uptake value (SUV_mean_) and xenograft-to-blood ratio of SUV_mean_. Regions of interest (ROI) were drawn for xenograft based upon ex vivo weight, assuming 1 g/ml tissue density. For blood pool measurements, a fixed-sized sphere was drawn in the center of the heart. After the final scan, mice were killed and organs of interest collected for biodistribution studies. Organs and standards of the injected tracer were counted in a calibrated well-type LKB-1282-Compu-gamma system (LKB WALLAC) and weighed. After decay correction, ex vivo tissue activity was expressed as the percentage of injected dose per gram tissue (%ID/g).

### Immunohistochemistry staining

For human CD103 IHC, previously formalin-fixed, paraffin-embedded tissue slices were deparaffinized in xylene and rehydrated. Heat-induced antigen retrieval was performed in 10 mM TRIS/EDTA (pH 9.0) at 100 °C for 15 min, endogenous peroxidase was blocked by 10-min incubation with 3% H_2_O_2_ in PBS and nonspecific binding of antibodies was blocked using 1% human serum albumin + 1% bovine serum albumin in PBS for 30 min. Next slides were incubated with rabbit anti-mouse CD103 antibody (1:500, ab224202, Abcam) for 60 min at room temperature. Incubation with secondary antibody (EnVision System, Dako HRP; Dako) was performed for 30 min, followed by application of diaminobenzidine chromogen for 10 min. Hematoxylin counterstaining was applied routinely. Digital scans of slides were acquired by a NanoZoomer 2.0-HT multi-slide scanner (Hamamatsu) and analyzed with NanoZoomer Digital Pathology viewer software.

### Statistics

Data are expressed as mean ± SD unless stated otherwise. Statistical analyses were performed in GraphPad Prism version 8.4.2 (GraphPad Software) using the Mann–Whitney test (2 groups, nonparametric).

### Supplementary Information

**Additional file 1: Fig. S1.** Tracer labeling (A) Optimization with different time points was assessed by instant thin-layer chromatography, where retardation factor of radiolabeled Fab fragment is 0.0 and complexed 68Ga is 1.0. (B) In the same manner was assessed amount of activity needed to achieve RCP >90%. (C-D) Purity and characterization of radiolabeled Fab fragment was done with SE-UPLC chromatograms using UV detection at 280 nm (protein) and radioactivity detector. Left panel shows chromatograms of unpurified reaction mixtures (Fab, 68GaCl and HEPES) and right panel – purified Fab by miniPD10 desalting column. (E) SE-UPLC chromatograms of hCD103.Fab01A conjugated with the chelator TFP-N-Suc-desferal-Fe using UV detection at 430 nm (Fe3+). (F) SE-UPLC chromatograms of [^89^Zr]Zr-hCD103.Fab01A using UV detection at 280 nm (protein) and radioactivity detector.

**Additional file 2: Fig. S2.** In vivo properties of CHO.CD103 and CHO.K1 cell lines.(A) Representative in vivo growth curves of CHO.CD103 (n=6) and CHO.K1 (n=4).(B) Representative ex vivo immunohistochemistry staining of CHO.CD103, CHO.K1 tumor xenografts and kidney.

**Additional file 3: Fig. S3.** In vivo [^89^Zr]Zr-hCD103.Fab01A and [^68^Ga]Ga-hCD103.Fab01A microPET imaging (A) Maximum intensity projection of coronal and sagittal [^68^Ga]Ga-hCD103.Fab01A PET scans, 3 hours post tracer injection in CHO.CD103 (top panel) and CHO.K1 xenograft mice (bottom panel). (B) MIP coronal [^89^Zr]Zr-hCD103.Fab01A PET scans, 3, 6 and 24 hours post tracer injection in CHO.CD103 (top panel) and CHO.K1 xenograft mice (bottom panel). Xenograft area marked with asterix.

**Additional file 4: Table 1.** Labeling characteristics of DFO-hCD103.Fab01 using gallium-68 and zirconium-89. **Table 2**. Recombinant molecules and antibodies.

## Data Availability

All data relevant to the study are included in the article or uploaded as online supplemental information.

## Abbreviations

^89^Zr: Zirconium-89
^68^Ga: Gallium-68
mAb: Monoclonal antibody
Fabs: Antibody fragments
PD-1: Programmed death-1
PD-L1: Programmed death-1 ligand
TILs: Tumour infiltrating lymphocytes
T_RM_-like cell: Tissue-resident memory-like phenotype
IHC: Immunohistochemistry
PET: Positron emission tomography
Df: TFP-N-Suc-desferal-Fe
VL and VH: Light and heavy variable domains
hCD103: Human integrin CD103
SUV_mean_: Mean standardized uptake value
TCA: Trichloroacetic acid
ROI: Region of interests
RCP: Radiochemical purity
SE-UPLC: Size exclusion ultra-performance liquid chromatography
PBS: Phosphate-buffered saline
