# Supplementary material for: Development of [89Zr]Zr-hCD103.Fab01A and [68Ga]Ga-hCD103.Fab01A for PET imaging to noninvasively assess cancer reactive T cell infiltration: Fab-based CD103 immunoPET
Source: EJNMMI Res. 2023 Nov 20;13:100. doi: 10.1186/s13550-023-01043-9 (PMC10661679; doi:10.1186/s13550-023-01043-9)
Supplement: Supplementary file 1 — Additional file 1: Fig. S1. Tracer labeling (A) Optimization with different time points was assessed by instant thin-layer chromatography, where retardation factor of radiolabeled Fab fragment is 0.0 and complexed 68Ga is 1.0. (B) In the same manner was assessed amount of activity needed to achieve RCP >90%. (C-D) Purity and characterization of radiolabeled Fab fragment was done with SE-UPLC chromatograms using UV detection at 280 nm (protein) and radioactivity detector. Left panel shows chromatograms of unpurified reaction mixtures (Fab, 68GaCl and HEPES) and right panel – purified Fab by miniPD10 desalting column. (E) SE-UPLC chromatograms of hCD103.Fab01A conjugated with the chelator TFP-N-Suc-desferal-Fe using UV detection at 430 nm (Fe3+). (F) SE-UPLC chromatograms of [89Zr]Zr-hCD103.Fab01A using UV detection at 280 nm (protein) and radioactivity detector. [file 13550_2023_1043_MOESM1_ESM.pdf]

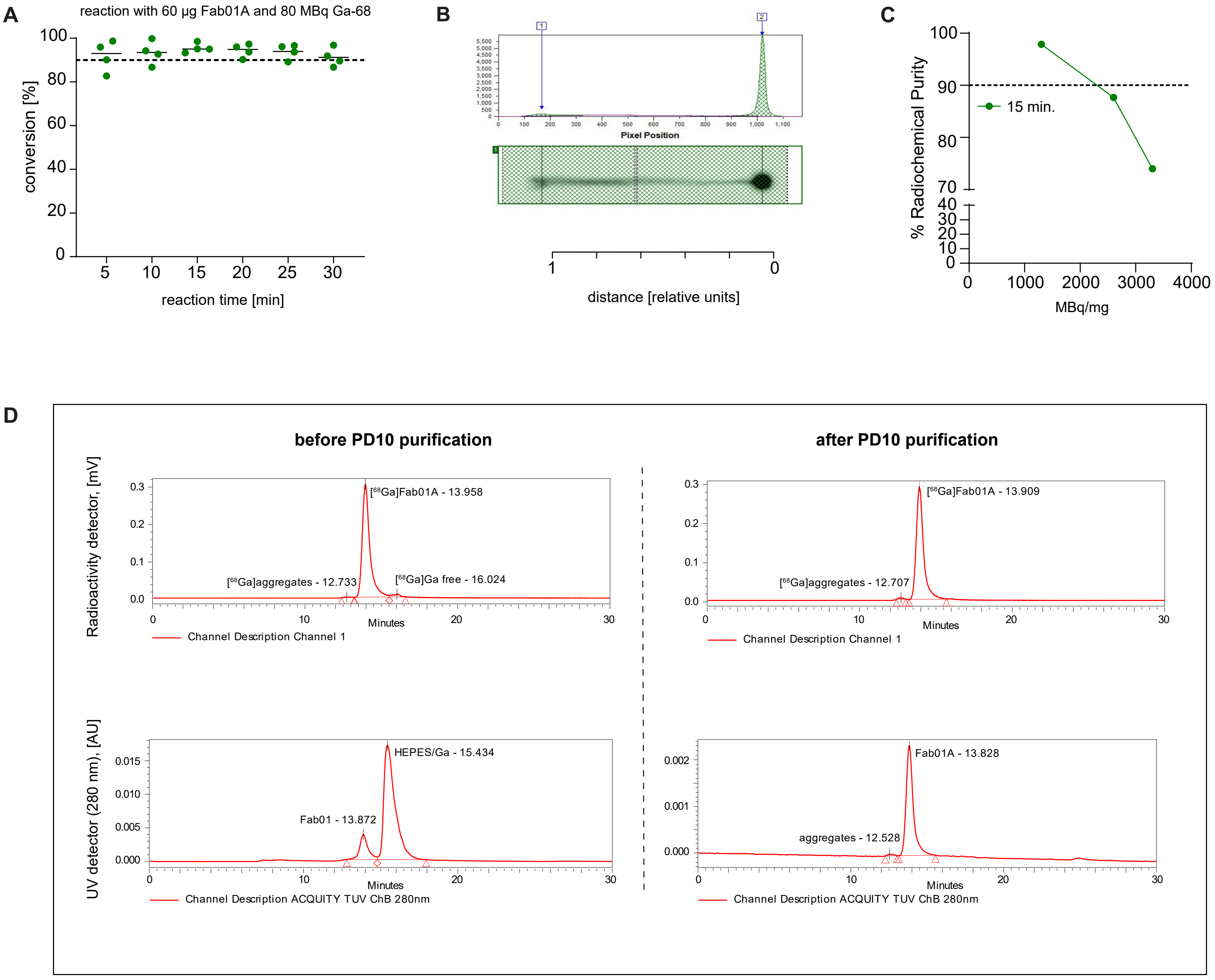

Supplementary Figure 1. Tracer labeling

A) Optimization with different time points was assessed by instant thin-layer chromatography, where retardation factor of radiolabeled Fab fragment is 0.0 and complexed 68Ga is 1.0

B) In the same manner was assessed amount of activity needed to achieve RCP >90%.

C-D) Purity and characterization of radiolabeled Fab fragment was done with SE-UPLC chromatograms using UV detection at 280 nm (protein) and radioactivity detector

Left panel shows chromatograms of unpurified reaction mixtures (Fab, 68GaCl and HEPES) and right panel – purified Fab by miniPD10 desalting column

E

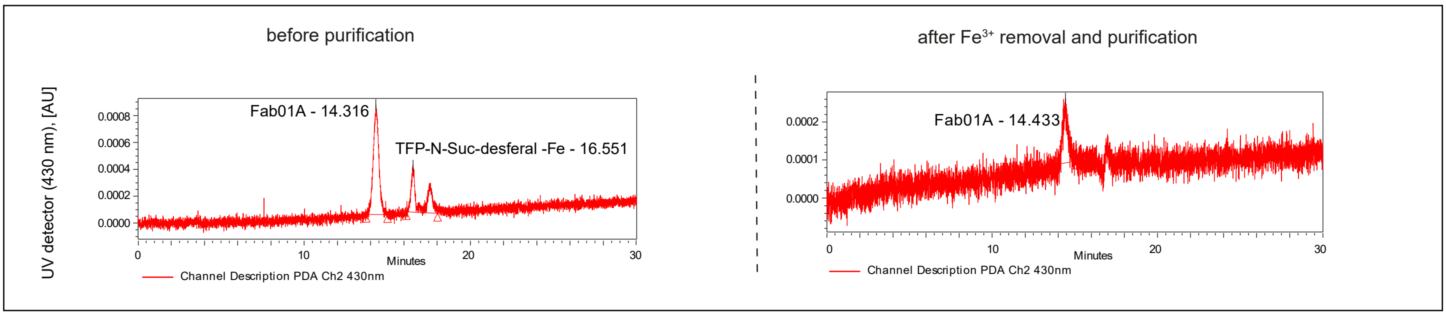

F

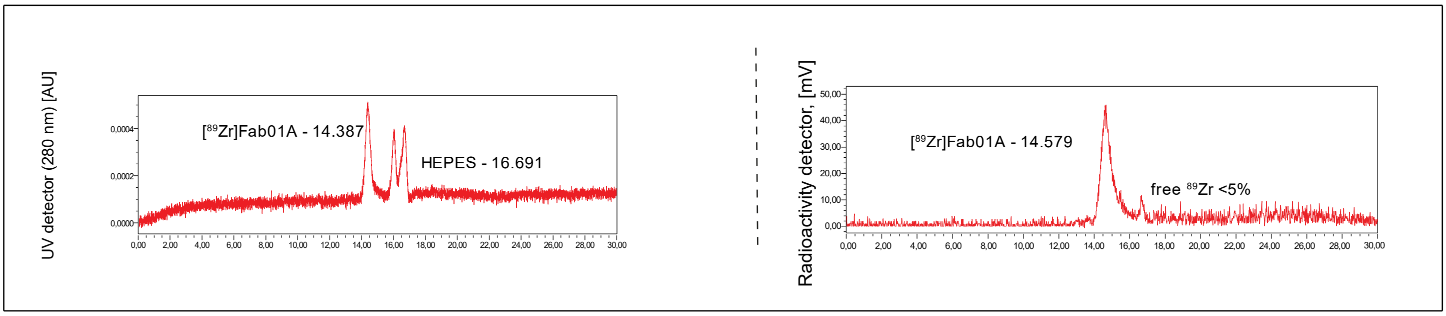

Supplementary Figure 1. Tracer labeling
E) SE-UPLC chromatograms of hCD103.Fab01A conjugated with the chelator TFP-N-Suc-desferal-Fe using UV detection at 430 nm (Fe3+).
F) SE-UPLC chromatograms of [89Zr]Zr-hCD103.Fab01A using UV detection at 280 nm (protein) at 280 nm and radioactivity detector
