# Supplementary material for: Development of [89Zr]Zr-hCD103.Fab01A and [68Ga]Ga-hCD103.Fab01A for PET imaging to noninvasively assess cancer reactive T cell infiltration: Fab-based CD103 immunoPET
Source: EJNMMI Res. 2023 Nov 20;13:100. doi: 10.1186/s13550-023-01043-9 (PMC10661679; doi:10.1186/s13550-023-01043-9)
Supplement: Supplementary file 2 — Additional file 2: Fig. S2. In vivo properties of CHO.CD103 and CHO.K1 cell lines.(A) Representative in vivo growth curves of CHO.CD103 (n=6) and CHO.K1 (n=4).(B) Representative ex vivo immunohistochemistry staining of CHO.CD103, CHO.K1 tumor xenografts and kidney. [file 13550_2023_1043_MOESM2_ESM.pdf]

Supplementary Figure 2

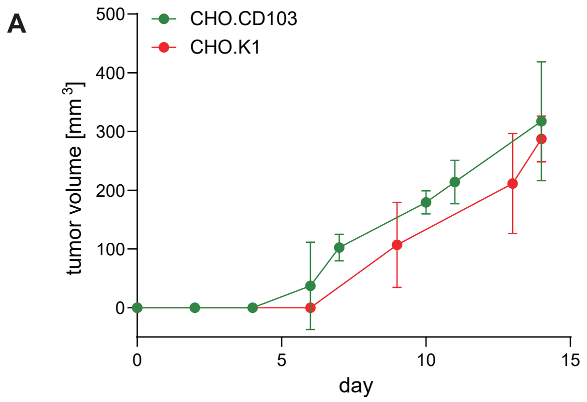

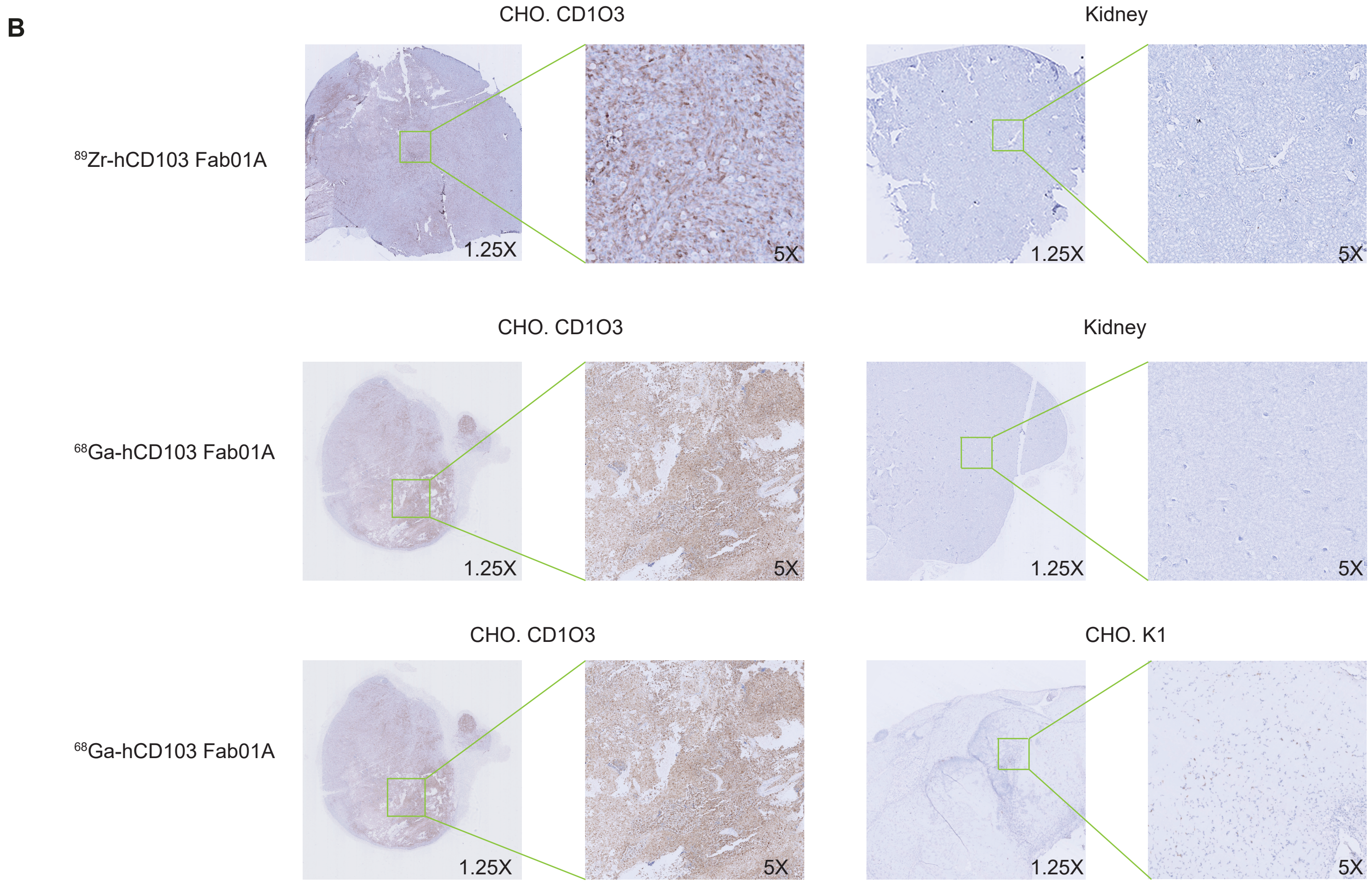

Supplementary Figure 2. In vivo properties of CHO.CD103 and CHO.K1 cell lines.
A) Representative in vivo growth curves of CHO.CD103 (n=6) and CHO.K1 (n=4).
B) Representative ex vivo immunohistochemistry staining of CHO.CD103, CHO.K1 xenografts and kidney.
