# Supplementary figures and images for: Development of [89Zr]Zr-hCD103.Fab01A and [68Ga]Ga-hCD103.Fab01A for PET imaging to noninvasively assess cancer reactive T cell infiltration: Fab-based CD103 immunoPET
Source: EJNMMI Res. 2023 Nov 20;13:100. doi: 10.1186/s13550-023-01043-9 (PMC10661679; doi:10.1186/s13550-023-01043-9)

**A** **$^{68}\text{Ga}$ -hCD103.Fab01A**

3h

CHO.hCD103

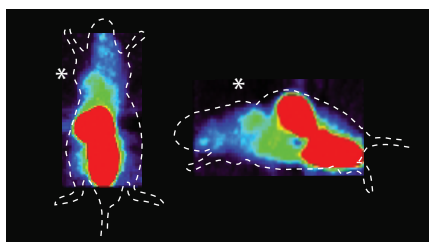

CHO.K1

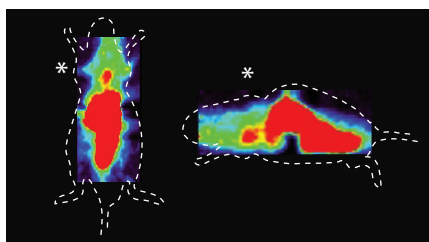SUV: 0 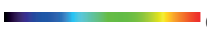 0.5**B** **$^{89}\text{Zr}$ -hCD103.Fab01A**

3h

6h

24h

CHO.hCD103

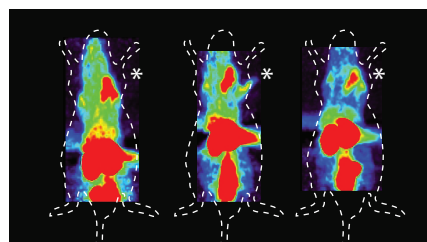

CHO.K1

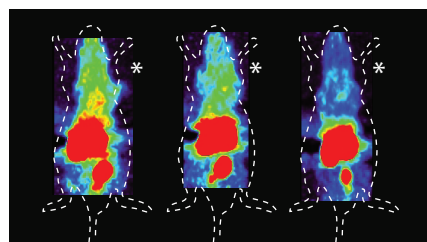SUV: 0 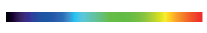 0.5

Supplement: Supplementary file 3 — Additional file 3: Fig. S3. In vivo [89Zr]Zr-hCD103.Fab01A and [68Ga]Ga-hCD103.Fab01A microPET imaging (A) Maximum intensity projection of coronal and sagittal [68Ga]Ga-hCD103.Fab01A PET scans, 3 hours post tracer injection in CHO.CD103 (top panel) and CHO.K1 xenograft mice (bottom panel). (B) MIP coronal [89Zr]Zr-hCD103.Fab01A PET scans, 3, 6 and 24 hours post tracer injection in CHO.CD103 (top panel) and CHO.K1 xenograft mice (bottom panel). Xenograft area marked with asterix. [file 13550_2023_1043_MOESM3_ESM.pdf]
