# Supplementary material for: Development of [89Zr]Zr-hCD103.Fab01A and [68Ga]Ga-hCD103.Fab01A for PET imaging to noninvasively assess cancer reactive T cell infiltration: Fab-based CD103 immunoPET
Source: EJNMMI Res. 2023 Nov 20;13:100. doi: 10.1186/s13550-023-01043-9 (PMC10661679; doi:10.1186/s13550-023-01043-9)
Supplement: Supplementary file 4 — Additional file 4: Table 1. Labeling characteristics of DFO-hCD103.Fab01 using gallium-68 and zirconium-89. Table 2. Recombinant molecules and antibodies. [file 13550_2023_1043_MOESM4_ESM.docx]

**Supplementary Table 1**. Labeling characteristics of DFO-hCD103.Fab01 using gallium-68 and zirconium-89.

| Radioisotope | Radiochemical purity [%] | Max. Apparent Specific Activity [MBq/µg] | Specific Activity  [GBq/mmol] |
| --- | --- | --- | --- |
| ^68^Ga | 95.0 ± 1.5^a^ | 1.1 ± 0.2 | 44 842 – 61 081 |
| ^89^Zr | 96.5 ± 0.4^b^ | 0.3 | 15 000 |

(^a^ radiochemical purity is determined as percentage of Fab-fragment-bound activity without purification by SE-UPLC; ^b^ radiochemical purity is determined as percentage of Fab-fragment-bound activity without purification by TCA assay.)

**Supplementary Table 2** Recombinant molecules and antibodies

| Anitbody/Reagents | Catolog number/clone | Vender |
| --- | --- | --- |
| CD103 (Integrin alpha E) Monoclonal Antibody | Ber-ACT8 | BD Biosciences |
| Recombinant Anti-CD103 antibody | ab224202 | Abcam |
| Dako Envision + System- HRP Labelled Polymer Anti-Rabbit | K4003 | Agilent/DaKo |
